# Group-tailored feedback on online mental health screening for university students: using cluster analysis

**DOI:** 10.1186/s12875-021-01622-6

**Published:** 2022-01-25

**Authors:** Seonmi Lee, Jiwoo Lim, Sangil Lee, Yoon Heo, Dooyoung Jung

**Affiliations:** 1 Department of Biomedical Engineering, Ulsan National Institute of Science and Technology, 50, UNIST-gil, Ulsan, 44919 Republic of Korea; 2 Hyperconnect, Seoul, Yeongdong-daero, Ulsan, Republic of Korea

**Keywords:** Mental health promotion, Online screening, Machine learning, eHealth, Primary care

## Abstract

**Background:**

The method by which mental health screening result reports are given affects the user’s health behavior. Lists with the distribution of scores in various mental health areas is difficult for users to understand, and if the results are negative, they may feel more embarrassed than necessary. Therefore, we propose using group-tailored feedback, grouping people of similar mental health types by cluster analysis for comprehensive explanations of multidimensional mental health.

**Methods:**

This cross-sectional, observational study was conducted using a qualitative approach based on cluster analysis. Data were collected via a developed mental screening website, with depression, anxiety, sleep problems, perfectionism, procrastination, and attention assessed for 2 weeks in January 2020 in Korea. Participants were randomly recruited, and sample size was 174. Total was divided into 25 with severe depression/anxiety (SDA+) and 149 without severe depression/anxiety (SDA-) according to the PHQ-9 and GAD-7 criteria. Cluster analysis was conducted in each group, and an ANOVA was performed to find significant clusters. Thereafter, structured discussion was performed with mental health professionals to define the features of the clusters and construct the feedback content initially. Thirteen expert counselors were interviewed to reconstruct the content and validate the effectiveness of the developed feedback.

**Results:**

SDA- was divided into 3 using the k-means algorithm, which showed the best performance (silhouette score = 0.32, CH score = 91.67) among the clustering methods. Perfectionism and procrastination were significant factors in discretizing the groups. SDA+ subgroups were integrated because only 25 people belonged to this group, and they need professional help rather than self-care. Mental status and treatment recommendations were determined for each group, and group names were assigned to represent their features. The developed feedback was assessed to improve mental health literacy (MHL) through integrative and understandable explanations of multidimensional mental health. Moreover, it appeared that a sense of belonging was induced to reduce reluctance to face the feedback.

**Conclusions:**

This study suggests group-tailored feedback using cluster analysis, which identifies groups of university students by integrating multidimensions of mental health. These methods can help students increase their interest in mental health and improve MHL to enable timely help.

**Supplementary Information:**

The online version contains supplementary material available at (10.1186/s12875-021-01622-6).

## Background

Young adults, including university students, experience immediate changes in their environments, leading to vulnerability to mental illness [1]. According to a Korean survey of university students’ recognition of mental healthcare services [2], 31.4% of students complained about severe stress, but over half of the students were not aware of how to receive healthcare services.

This indicates that students have a low ability to know when and where to seek help and develop competencies designed to improve their mental health and self-management capabilities [3, 4]. This ability is called mental health literacy (MHL). Low MHL inhibits early detection, preventing the success of intervention, and causes severe mental problems [1, 5]. To increase MHL, various kinds of interventions have been studied. For adolescents, educational interventions in school have appeared effective [6]. However, it is not easy for university students to join the educational system, as it is time consuming [7]. Another way to increase MHL is to improve problem recognition through mental health screening [8, 9]. Mental health screening intervention with appropriate feedback can save time and increase individuals’ interests in mental health, so many trials have attempted to develop practical online screening tools [10–13].

In the case of screenings that provide feedback in person at institutions such as medical facilities or counseling centers, experts deliver the results based on the patients’ mental conditions and characteristics [8, 14]. On the other hand, no one appropriately explains the student’s mental health in the case of online screening. For this reason, the means by which screening feedback is provided plays an essential role in motivation for treatment and self-reflection [15]. Feedback with a simple numeric and general description about severity is not efficient in increasing self-reflection or a willingness to develop one’s mental health [16]. Therefore, it is necessary to study tailored feedback that communicates an individual’s status and provides helpful directions.

Nonetheless, there are few studies on the feedback methods. The most widespread method of online screening is person-tailored feedback, which provides the result individually by generating algorithms to set cutoff points and distinguish severity accordingly [17]. In this case, it is easy to define and explain information according to the severity of each mental problem, such as mild, moderate, or severe, but it is challenging to give comprehensive explanations that account for multiple mental aspects jointly. For example, on a website that screens mental health online and suggests CBT-style treatment, people can select mental diseases they want to screen for but receive the screening consequences of each disease separately [18]. A study found an algorithm to examine the severity of depression with its causes that tried to categorize people by fixed conditions [19]. Another tried to screen depression and anxiety and followed the treatment steps of each questionnaire to accurately determine one’s mental status [20]. However, since mental diseases have high comorbidity, it would be helpful to increase one’s MHL and empathy if a variety of symptoms are considered together to provide recommendations.

The feedback method generally used is person-tailored feedback which can satisfy the desire for self-knowledge by displaying results objectively and accurately and can help prevent deterioration in mental health. On the other hand, it can aggravate negative thoughts and isolation in people suffering from severe depression or anxiety if the results indicate a severe condition [21]. According to [22], it has been discovered that perceived belongingness and social support in society helps people who suffer from severe depression reduce stress and feel empathy. This shows that it is necessary to find method other than individual and direct reporting to measure such belongingness or supportive factors.

Therefore, the established person-tailored feedback method has limitations in that it cannot determine overall mental aspects or increase the sense of social belongingness and support that can positively affect people with severe problems. From this context, we suggest group-tailored feedback using cluster analysis. First, we divided the population into specific groups based on the overall characteristics of university students’ mental health using cluster analysis. Then, we tried to identify the group each individual belongs to in order to elicit bonds and empathy and reduce stress to enable the individual to better face their current mental status.

In this context, this paper aims to develop a method for providing group-tailored feedback through online screening services for mental health based on the multidimensional mental status of university students.

## Methods

### Study design

**Fig. 1:**
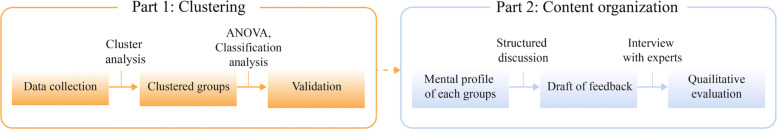
Process to develop group-tailored feedback using cluster analysis

This study is a cross-sectional, observational study conducted using a qualitative research approach based on cluster analysis. The overall procedure consists of two parts: *clustering* and *content organization*. In the *clustering* part, a cluster analysis of the mental health of university students was conducted. We initially searched for studies on features of mental health in university students and selected mental health dimensions (MHDs) that could represent those features. The score for each MHD was collected online and utilized for cluster analysis to assign groups. In the *content organization* part, the appropriate content of the feedback was determined. The elements of the content were extracted using a structured discussion based on the results of the clustering part. To evaluate the content and its effect on user recognition of mental health, we drafted the feedback with the elements found in the structured discussion, and the developed feedback was reviewed by professional counselors. The overall process is shown in Fig. 1.

### Part 1: clustering

#### Questionnaires/Inventories

To estimate overall mental health, we conducted a literature review of the common mental problems and risk factors of university students. Accordingly, we selected MHDs and found inventories to assess them.

Depression and anxiety were discovered to be highly prevalent psychiatric problems among university students, with 20% of students experiencing those diseases [23, 24]. In particular, it was found that depression is the most common mental disease among Korean college students suffer from, and is directly related to feelings of defeat, and suicidal thoughts [25].

Sleep disorder is another problem that university students commonly experience. Sleep disorder has high comorbidity with anxiety and depression [26]. Even if there are no severe psychiatric problems, sleep problems can occur because of irregular lifestyles caused by academic burden [27].

Unlike individuals in other stages of life, academic performance is a significant factor in the defective mental health of university students [28, 29]. Stress over academic performance and fear about future careers are related to perfectionism [30, 31].

Perfectionism has positive effects on academic performance through adaptive perfectionism, which increases self-esteem and conscientiousness [32]. However, perfectionism is associated with high personal standards, and striving to achieve those standards can lead to stress and mental problems such as anxiety [33]. Maladaptive perfectionism is largely related to such mental diseases. This kind of perfectionism is not self-oriented but socially prescribed via the expectations of parents or critics [34]. Because of these traits, people with maladaptive perfectionism have many concerns, including worries rather than self-esteem, and this trait can be a risk factor for anxiety and depression [35]. In addition, these traits are associated with procrastination. Students want their work to be perfect, but fear of failure followed by high expectations leads to procrastination [36].

Attention-deficit/hyperactivity disorder negatively affects academic performance [37]. This kind of attention disorder can lead deterioration of academic performance increase stress and contribute to low mental health.

Consequently, we selected six MHDs of university students: depression, anxiety, sleep problems, perfectionism, procrastination, and attention problems. Existing psychological questionnaires were utilized to assess each aspect. The Patient Health Questionnaire-9 (PHQ-9) is a 9-item questionnaire that evaluates depression on a range from 0 to 27 [38]. The Generalized Anxiety Disorder-7 (GAD-7) was utilized to identify probable cases of generalized anxiety. The total GAD-7 score is the sum of scores for the 7-item questionnaire and ranges from 0 to 21 [39]. The Adolescent Sleep Hygiene Scale (ASHS) includes 33 items that assess sleep hygiene practices in several conceptual domains [40]. In our study, we used the reverse score of the ASHS to estimate sleep problems. To check concentration level, we used the Adult ADHD Self-Report Scale (ASRS) [1]. The ASRS comprises Part A and B. Six items in part A were used to estimate the probability of adult ADHD, while part B consists of checklists of symptoms. Therefore, we used only Part A to score concentration. The Frost Multidimensional Perfectionism Scale (FMPS), which consists of 35 items measuring both self-oriented perfectionism and socially prescribed perfectionism, was utilized to determine overall perfectionism [34]. The Aitken Procrastination Inventory (API) was developed to identify procrastinators among college students in a previous study [41]. The API includes 19 items and results in a total score ranging from 19 to 95.

#### Data collection

Participants for the survey were randomly chosen from 4300 students in Ulsan National Institute of Science and Technology (UNIST) in Korea and were invited to participate through an invitation email to visit the developed website. We developed a web-based survey with the selected questionnaires for data collection. On the first page, the purpose of the data collection was stated. The informed consent to provide their screening results and simple demographic information, such as degree course, sex, and age, without personal identifying information, was obtained from all participants. For recruitment, each student at UNIST received an e-mail inviting them to visit the website. The participants voluntarily visited the website to check their mental health. Data were collected for 2 weeks in January 2020.

Our sample comprised 174 undergraduate and graduate students. A total of 189 students participated in the survey, but 15 participants (12.4%) who answered the redundant questions differently were excluded. The mean age of the sample was 24 (*SD*=3.2) years, and there was a small difference in the proportion of males (45%) and females (55%).

The demographic characteristics of the participants are shown in Table 1. The distribution of scores for each dimension is shown in Additional file 1. After collecting the score of each mental aspect, we compared them with the cutoff points for clinical status. The PHQ-9, GAD-7, and ASRS have cutoff points to distinguish clinical status. However, the FMPS, API, and ASHS estimate tendencies without a determined standard, so a higher score of each represents higher perfectionism, procrastination, and sleep hygiene, respectively. The distributions of FMPS (*M*=103.9,*SD*=17.4), API (*M*=53.7,*SD*=7.8) and ASHS (*M*=4.62,*SD*=0.6) scores from previous studies were similar to those collected here [42, 43]. The data collected for those dimensions were normally distributed with a z-score of kurtosis and skewness less than ∣2.1∣.

**Table 1:** Demographics of participants

Education	Gender	Year *M*(*SD*)	n
Undergraduate	Female	22.5 (1.4)	48
	Male	22.0 (2.0)	33
Graduate school under 4 semesters	Female	25.6 (2.0)	23
	Male	26.0 (2.7)	20
Graduate school over 4 semesters	Female	27.2 (1.6)	29
	Male	28.3 (3.4)	21
Total		24.0 (3.2)	174

#### Cluster analysis

We performed cluster analysis with the MHD scores collected by online screening to group individuals with similar mental states. Initially, we divided the total number of people into a group with severe depression/anxiety (SDA+) and another without severe depression/anxiety (SDA-) to distinguish those who need immediate treatment by professionals to recommend counseling. The participants with a PHQ-9 or GAD-7 ≥ 15 were labeled SDA+, and the rest were labeled SDA-.

After precategorization, cluster analysis was executed in each group. According to previous studies, cluster analysis helps determine specific features of latent groups of people with similar mental diseases [44, 45]. Therefore, we used cluster analysis to define latent types and discover unknown subgroups. Six kinds of clustering algorithms were optimized: k-means, k-medoids, agglomerative nesting with linkage of ward (ANW), agglomerative nesting with linkage of average (ANA), mean shift (MS), and DBSCAN. Then, we used the best performing model for clustering. Each model requires parameters to determine the number of clusters. We adjusted the desired group (k) for k-means, k-medoids, ANW, ANA, and bandwidth of kernel function for MS. Finally, we adjusted radius eps for DBSCAN.

The silhouette score and Calinski-Harabasz score (CH score) were utilized to find optimized parameters for the models and compare robustness. The silhouette score is calculated by the mean distance between a sample and other data points in a cluster and the mean distance between the sample and the data points in other clusters [46]. The CH score is defined as the ratio of the between-cluster dispersion mean and the within-cluster dispersion [47]. According to a previous study, the CH score is the most effective standard for comparing clustering performance [48]. Therefore, we considered primarily the CH score and silhouette scores to select the best performing algorithm. A machine learning toolbox named Scikit-learn was used in this process.

#### Validation

To evaluate the differences among groups with MHD variances, we used analysis of variance (ANOVA) statistical analysis. ANOVA represents the degree to which each factor affects clustering. Because cluster analysis was performed after bisecting the data into SDA- and SDA+, the ANOVA was conducted separately. Then, we utilized classification techniques for the two groups independently to validate whether the groups were well distributed to classify new data correspondingly. Initially, we labeled each dataset with its subgroups and split them into training and testing sets at a ratio of 7:3. Then, the accuracy and area under the ROC curve (AUC) were calculated to evaluate the classification performance. Accuracy is defined by the sum of correctly classified data divided by the number of total data points. The AUC is used to evaluate the performance of a diagnostic tool and can be described as an integration of sensitivity by all possible values of specificity. The following classifiers are used: logistic regression, support vector machine with radial basis function (SVM), k-nearest neighbors (KNN), and decision tree. The process was implemented using the Scikit-learn toolbox in Python 3.8.8.

### Part 2: content organization

#### Structured discussion

To organize the features of subgroups and recommendations for mental healthcare, a structured discussion was used. The participants were a psychiatrist who was the corresponding author and a psychologist in the same laboratory, with the first author acted as the moderator. They were introduced to the theme of the research and how the cluster was derived. Displaying the mental profiles in each subgroup, the topic “How can you express those groups and give recommendations for them?” was given. The moderator asked for findings or insights for each case, and the discussion was open to allow all ideas that the participants mutually agreed upon to be obtained. Agreement of recording was achieved previously. The discussion was transcribed after recording, and a narrative summary of the findings was completed. Based on the findings, the first draft for group-tailored feedback was designed.

#### Interview with experts

To evaluate how group-tailored feedback affects recognition of mental health, a brief interview was conducted with university counselors. A total of 13 individuals with Mental Health Clinical Psychologist Certificates from the Korean Counseling Psychological Association participated. The interview was conducted for an hour, starting with a brief explanation of the study. The first part evaluated the validity of the feedback developed in this study. In this part, we showed the draft of group-tailored feedback constructed by the structured discussion and asked whether the explanation for each group was reliable and whether the recommendations were appropriately suggested. In the second part, several questions were asked to identify the distinctive impact of group feedback compared to general feedback. The sample of general feedback for comparison was produced to show the score of each MHD and its explanation individually, as in the existing methods, to show the results. In addition, the elements of the content provided by group feedback were included in the sample similarly to make the content as similar as possible. Initially, the strengths and weaknesses of the two kinds of feedback were queried with open questions. In the end, the participants were asked to estimate the amount of change in recognition of and interest in mental health, motivation to improve mental health, and fear of confronting one’s mental health. They answered subjectively and then checked the effect of each type of feedback on 5-Likert scales. Finally, we utilized the Wilcoxon signed-rank test to statistically evaluate the difference between the two methods.

The study was approved by the Ulsan National Institute of Science and Technology Institutional Review Board (UNISTIRB-21-07-A) and was conducted in accordance with the principles of the Declaration of Helsinki.

## Result

### Cluster analysis

The majority of the sample (85.6%) was classified as SDA-, and the rest (14.4%) were classified as SDA+. The overall distributions of mental aspects in each group are shown in Additional file 1. The scores of all MHDs in the SDA- group were lower than those in the SDA+ group.

**Fig. 2:**
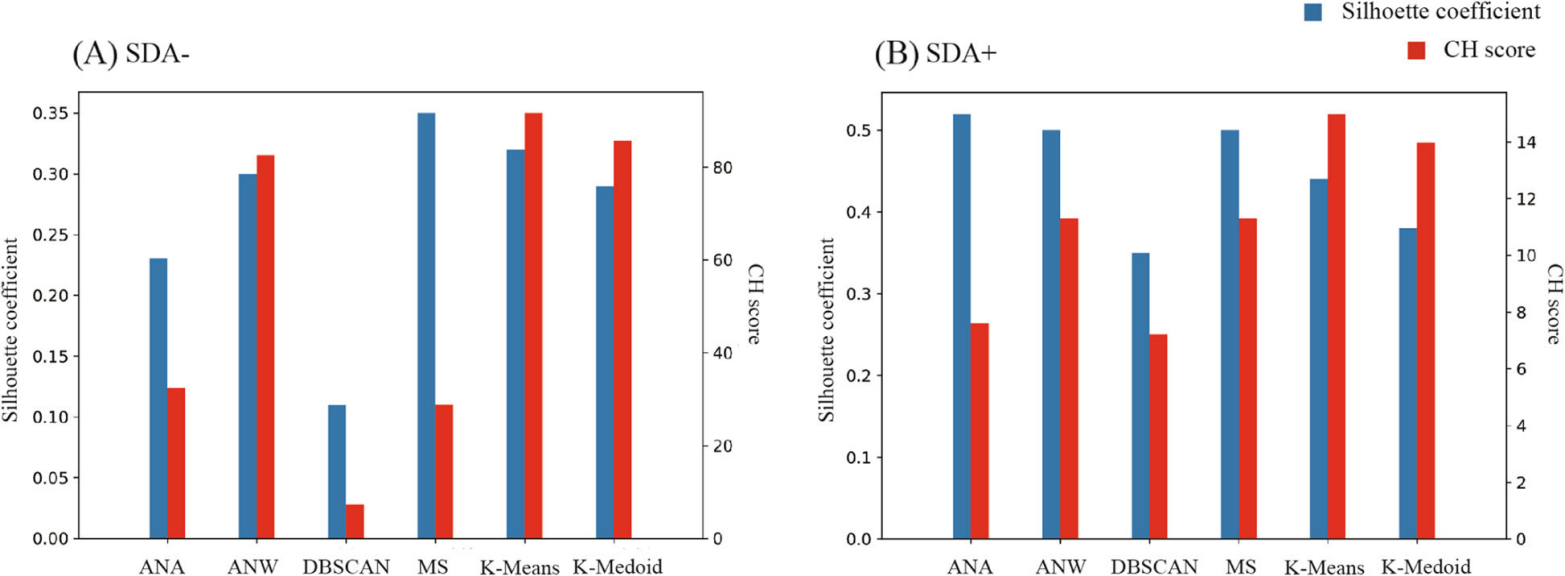
Silhouette score and CH score of each cluster model in (**A**) SDA- and (**B**) SDA+

We found optimal parameters for different models, and the details are shown in Additional file 2. We compared the optimized models to select the best performing model, which shows the highest separation of clusters according to silhouette score and CH score. According to Fig. 2, k-means clustering showed the highest CH score with a relatively high silhouette score for both SDA- (CH score = 91.67, silhouette score = 0.32) and SDA+ (CH score = 14.99, silhouette score = 0.44), indicating that the k-means showed the best performance in distributing the data, so we utilized k-means for cluster analysis in our study. Using k-means, we found 3 clusters in SDA- and 2 in SDA+.

#### Clusters of SDA-

**Fig. 3:**
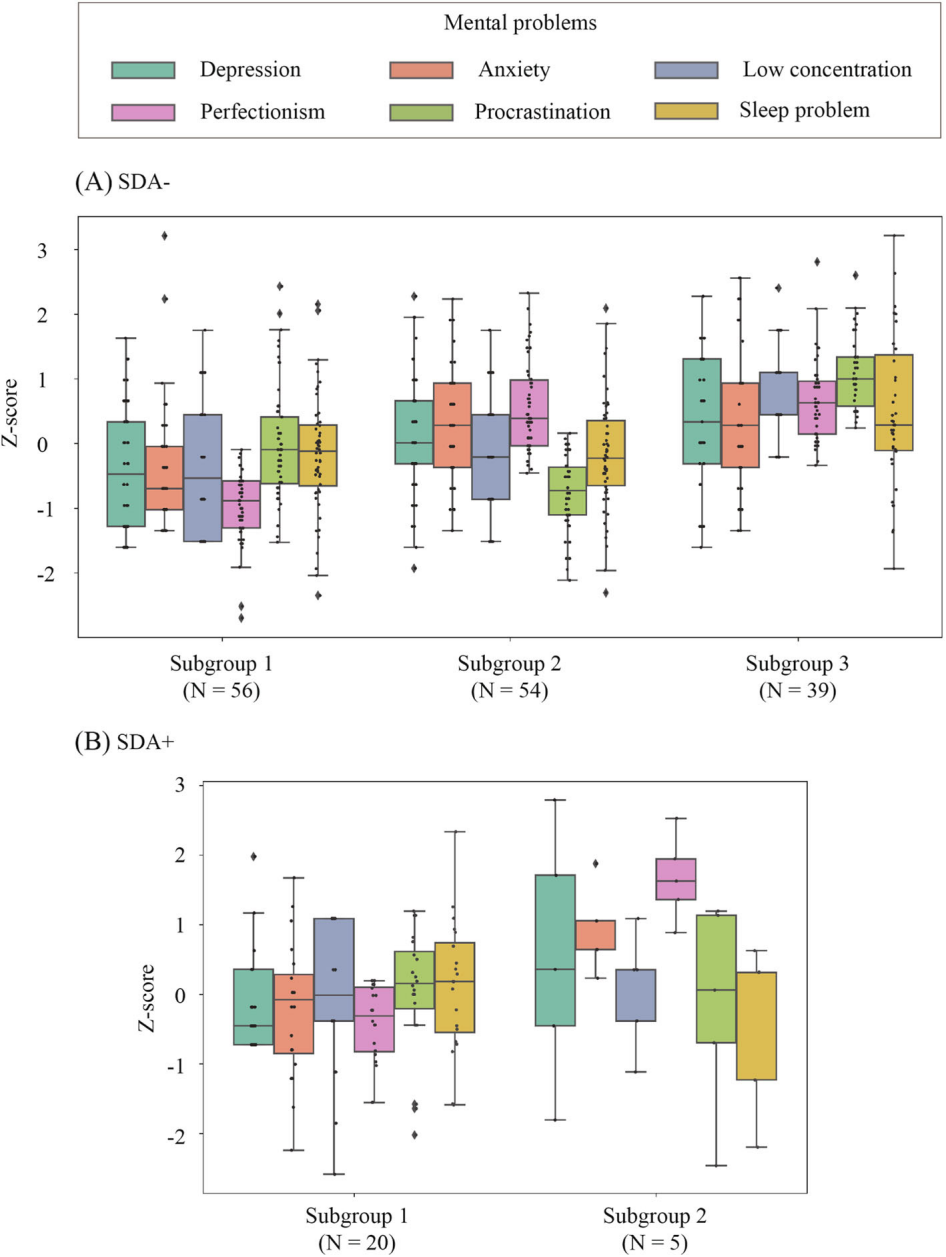
Score distribution of sub-groups divided by K-means clustering in (**A**) SDA- and (**B**) SDA+

Three subgroups in which people had similar mental health features were formed in SDA-. We conducted a z-score transformation for the scores of each mental aspect to compare the tendency of each group. Figure 3 shows the z-score for each subgroup. Subgroup 1 showed a relatively low score distribution in overall properties compared with the scores of the other subgroups and showed a decrease of 1 *SD* in procrastination from the scores of Subgroup 2. This implies that people in this group had comparatively stable mental health. It occupied 37.6% of SDA-. Subgroup 3, including 26.2% SDA-, presented a contrasting tendency to Subgroup 1. They had relatively poor mental health, based on the results of the remaining subgroups. Notably, Subgroup 3 showed an approximately 1.5 *SD* increase in perfectionism compared to Subgroup 1 and in procrastination compared to Subgroup 2. Finally, Subgroup 2 showed relatively less procrastination in the workplace and more perfectionistic standards (36.2%) This group showed slightly higher anxiety and depression levels than Subgroup 1. According to the difference in the medians of each mental aspect, differences between subgroups prominently appeared in perfectionism and procrastination. From the perspective of perfectionism and procrastination, three subgroups had distinctive features: low perfectionism with a small amount of procrastination, high perfectionism with low procrastination, and high perfectionism with high procrastination.

#### Clusters of SDA+

There was a minority of people (*N* = 25) in the SDA+ group, but they were divided into two groups. Figure 3 shows that the median score of perfectionism varied significantly, with a 1.5 *SD* variation between the two subgroups. A mild difference in anxiety level appeared to separate the subgroups in SDA+. As a result, Subgroup 1 showed lower perfectionism and anxiety levels than Subgroup 2.

### Validation

To evaluate the variability of each mental aspect among the divided groups, an ANOVA was conducted for the SDA- and SDA+ each (Table 2). In SDA-, all MHDs affected subgroup division, but the *F*-value of perfectionism was highest among them. In SDA+, perfectionism and anxiety were significant factors in dividing the subgroups, but other MHDs seldom affected the group division. From both results, perfectionism was revealed as a critical criterion for dividing the subgroups.

**Table 2:** Variability of six MHDs among clustered groups by ANOVA *F* test

MHD	SDA-		SDA+	
	*F*	*P*	*F*	*P*
Concentration	24.89	< 0.01**	0.02	0.89
Perfectionism	112.82	< 0.01**	52.92	0.01**
Procrastination	78.15	< 0.01**	0.14	0.72
Sleep problem	7.39	< 0.01**	1.14	0.3
Depression	10.2	< 0.01**	1.66	0.2
Anxiety	7.53	< 0.01**	5.7	0.03*

* *p*<.05, ** *p*<.01

To confirm that the clustering result was significant for classifying the new data, we evaluated the performance of various classification models with labels from cluster analysis. We found that the AUC was over 0.9 regardless of the classifier type (Table 3). This means that the results of the cluster analysis could divide each group with more than 90% accuracy, regardless of the training model. Additionally, the average accuracy in both SDA- and SDA+ was higher than 0.9.

**Table 3:** Comparison of performance of various classification models

Classifier	SDA-		SDA+	
	AUC	Acc	AUC	Acc
Logistic regression	0.92	0.84	1.0	1.0
SVM	1.0	0.96	1.0	0.76
KNN	0.99	0.93	1.0	0.94
Decision tree	0.93	0.95	1.0	1.0
Average	0.96	0.92	1.0	0.93

### Structured discussion

We found the elements of the group-tailored feedback content through structured discussion. The findings are divided into three categories: subgroup features and recommendations in SDA-, subgroup features and recommendations in SDA+, and overall considerations for group-tailored feedback content in online mental health screening.

#### Subgroup features and recommendations in SDA-

Individuals in the SDA- group do not need to obtain help from professionals immediately. Instead, it is necessary to provide clear information on their strengths and difficulties they may experience based on their classification type. Perfectionism, which can motivate increasing performance and stress when an outcome does not meet expectations, is a critical factor in separating the three subgroups.

Subgroup 1 showed lower scores on most dimensions than the other groups while showing slight procrastination, so their mental health was described as ‘stable’ and ‘leisurely’. For this reason, the group was named ‘Yuyujajoek (hereinafter the leisurely)’, which means feeling free and peaceful. However, this does not indicate that they have no worries. Accordingly, it is recommended that they be able to visit the school counseling center voluntarily if they have problems in their daily lives related to decision making for their future careers, dating, or other daily issues. It is essential to inform them that they can obtain help openly for challenging problems in the future.

In contrast, Subgroup 3 showed relatively high scores in overall dimensions compared with the other two subgroups. As a strength, they did not exhibit current clinical levels of emotional stress. Nevertheless, they have a high level of procrastination and strong perfectionism, which indicates that they cannot satisfy high expectations to offset their worries. This phenomenon transcends attention problems, so performance is affected. The primary recommendation is to reduce procrastination, which can cause stress through interaction with intense perfectionism. Therefore, maintaining a routine life is required, and the use of applications to set and achieve practical goals is encouraged. Behavior to release stress, such as meditation and walking, is useful for those in this subgroup. They are recommended to obtain help if their worries or stress become uncontrollable. This subgroup was called ‘Geureun Wanbyeokjui (hereinafter the lazy perfectionist)’.

Subgroup 2, with a high level of perfectionism and low procrastination, showed the ability to maintain a ‘daily routine’, with a routine sleep time indicated by a stable sleep hygiene score. However, they are vulnerable to the stress caused by perfectionism. An interviewee said, *“They manage their stress according to the level of depression and anxiety, but they can get excessive stress when the routine life cannot be maintained.”* Previous studies have found that people being aware of their perfectionism and that it can cause stress has a significant effect on relieving stress, and strategies to cope with stress help [33, 49]. Therefore, it is recommended to help these individuals receive an explanation of how perfectionism works and practical strategies for coping with stress. This group tends to have a routine life and has a relatively stable mood with some extent of perfectionism; thus, it was named ‘Geonganghan Wanbyeokjui (hereinafter the healthy perfectionist)’.

#### Subgroup features and recommendations in SDA+

The SDA+ subgroups were integrated because there were few people in each group, and the members of these groups required help beyond doing something for themselves. People in this group are expected to have clinical symptoms because of emotional problems. Nonetheless, their status should not be communicated directly, as it can lead to negative stigma. Describing the status of these individuals using ‘vulnerable’ rather ‘bad’ and adding ‘it can be temporary’ can help reduce unpleasant or disagreeable responses.

Additionally, interviewees added that explaining the result in terms of group profiles can result in solidarity not experienced following individual feedback. This group was named ‘Maeumari (henceforth the disturbed mind)’, which means having sickness of the mind but can reduce negative nuance of mental illness.

#### Overall considerations for feedback

There are three cautions that should be expressed when providing group-tailored screening feedback. First, it is crucial to clarify that the results can be temporary. This element was emphasized through overall discussions of the SDA- and SDA+ content. This clarification will help SDA+ individuals obtain the *“possibility for change”* and SDA- individuals to exhibit consistent help-seeking behavior related to MHL. Second, careful empathy is needed through the use of expressions such as ‘may’, ‘be likely to’, ‘seems like’, and ‘tend to’. Finally, all the feedback should be presented with encouraging factors for improvement.

Based on these findings, we designed a group-tailored feedback page to conduct interviews with other mental health or psychology experts to verify the findings (Additional file 3).

### Qualitative evaluation

We conducted interviews to evaluate the practicality and effect of the developed group-tailored feedback. Initially, the interviewees evaluated the content of the drafts created based on the structured discussion. Thereby, they explained the effect of the group-tailored feedback based on the survey results with Likert scores at the end of the interview.

#### Contents evaluation

##### Evaluation of naming

The group name is important in group-tailored feedback because it implies the group’s characteristics and encourages interest among the students. In this interview, half of the participants indicated that the name was interesting and expressed the group’s characteristics well (R1,4,5,9,11,12). Avoiding negative connotations and emphasizing positive aspects of the group were important criteria in evaluating the name of each group. For example, ’the healthy perfectionist’ has a positive meaning, with the word ’healthy’ delivering relief to students who cannot satisfy themselves easily because of perfectionism. Additionally, ’Maeumari’, the Korean name of ’the disturbed mind’, was able to give a warm feeling to reduce reluctance.

*“People included in the ’healthy perfectionist’ group are usually self-aware and tend to compare themselves with others, so they may not see themselves positively. However, it seems to tell me that this can be healthy”* (R1)

*“I think this group name is the best in that there is no negative connotation. I think students will have less reluctance to face the result.”* (R9)

##### Evaluation of mental health features and recommendations

The feedback is divided into two parts: the mental health features of each group and health recommendations. For the mental health features of the group, it was noted that all groups except ‘the lazy perfectionist’ group should be provided with detailed descriptions. A detailed explanation of the strengths and weaknesses of the group based on the six MHDs examined in the previous questionnaires helps participants empathize and engage. In particular, emphasizing strengths by explaining them in detail appears necessary to increase the acceptability of mental health recommendations.

On the other hand, the features of ‘the lazy perfectionist’ group were noted to be adequately reflected because the explanation of perfectionism included both strengths and weaknesses. The explanation of perfectionism could allow people to better understand and accept their current status (R2,5,7,8,9).

In this respect, it was recommended that the explanation of perfectionism be added to ‘the healthy perfec- tionist’ group to help the individuals understand their state (R1,7,13). The strengths and weaknesses of this group were included adequately, but it was necessary to describe its strengths more specifically based on MHDs (R 1,2,5,8,9,11). It is expected that this would make readers comfortably accept their shortcomings.

*“If you talk about the positive part a lot, the student can admit (result) and think (in the part where you can get stressed) ‘Oh yeah, I can feel much pressure, so I think it would be good to receive advice.”’* (R9)

In the case of ‘the leisurely’, it was difficult to describe the characteristics, as the scores generally tended to be stable (R1,3,8). Therefore, it was suggested to refer to the questionnaires of the scale to specify the strengths (R2,4,6,8,11,13). The remarkable feature of this group was that the level of procrastination for academic work was higher than that of ‘he healthy perfectionist’ group. R3,4,6 indicated that the people in this group could experience less real achievement than ‘the healthy perfectionist’ because they experience less tension when working.

For ‘the disturbed mind’, it is necessary to explain the emotions they can experience in relation to daily discomfort rather than emphasizing depression and anxiety severity (R2,5,8,12).

*“These friends are depressed now, but they don’t accept the depression, saying that all of these parts will be okay. In that case, if you tell them about everyday discomforts because you are depressed, they will be able to get counseling for their emotional problems.”* (R5)

Unlike for the description of mental health features, the recommendations for all groups except ‘the leisurely’ group were evaluated as appropriate. The stress coping method for ‘the healthy perfectionist’ group and the link to counseling services and additional questionnaire on emotional stress for the ‘the disturbed mind’ group were evaluated as appropriate (R1,3,5,6,8, 9,10,11,12). The interviewees noted that emphasizing a regular life for ‘the lazy perfectionist’ is also helpful, but setting a small goal as an example of a regular life was preferred (R8,9,11,12). Since people in this group tend to establish plans perfectly and feel hard to achieve, it was necessary to give a simple example that can be easily carried out.

The interviewees considered that the recommendations for the ‘the leisurely’ group were not associated with the particular features of the group. Instead, establishing specific goals was recommended to compensate for the lack of realistic achievement the people in this group usually have (R3,4). In addition, the people can fixate on the results and misunderstand them as indicating fixed characteristics. For the solution, continuous monitoring was recommended (R8,10).

*“Because it is a result that can change at any time when stress increases, it would be nice to mention continuous checking and to manage their mental health.”* (R8)

##### Evaluation of expressions

In addition to mental health features and recommendations, several expression methods for feedback are important in indicate the students’ empathy and understanding. First, when choosing a word, it is best to use universal and everyday language to help students communicate clearly and avoid heavy words, as it may cause misunderstandings (R1,2,4,7,8,11,12). For example, the preferred expressions include ‘difficulties in the mind’ rather than ‘mental problems’ and ‘discomfort in awareness’ rather than ‘cognitive discomfort’.

Second, it is possible to minimize the discrepancy caused by deviations within the group by using expressions indicating that the result is relative and avoiding a definitive word. The preferred expressions included ‘based on the average’, ‘compared to’ or ‘there may be a tendency to’ (R5,10,11) because, if the results are deterministically explained, it may be difficult for the students at the far ends of the distributions to accept the results.

Third, it is necessary to indicate that the results do not indicate fixed characteristics such as personality types (R1,5,8,10,12).

Last, to increase user acceptance, it is necessary to use sympathetic examples. The presentation of the problems that may be experienced according to the mental status of each group was well received by the interviewees and was considered an essential factor that could arouse empathy and acceptance (R4,9,12).

*“Friends who score far from the mean in the distribution may feel a little uncomfortable. However, if we use relative expressions and indicate that the result is the average in the group, it will be adjusted.”* (R5)

*“If I group them, I think students can mistake them for my fixed characteristics like MBTI(Myers-Briggs Type Indicator) because our state of mind is not constant; it changes as we live.”* (R10)

#### Evaluation of the effect of group-tailored feedback

In the interview, we investigated the effect of group feedback compared to general feedback. In this part, the opinions of the interviewees were investigated. The survey with the Likert scale was analyzed with the subjective responses to strengths and weaknesses of the group-tailored feedback. The results of the survey are shown in Table 4. As a result, group feedback was significantly more effective in producing an understanding of and concern for mental health and significantly decreasing resistance to confronting one’s mental status that individuals may feel when they receive poor results.

**Table 4:** 5-point Likert scale evaluation of the effect of general feedback and group feedback and Wilcoxon signed-rank test result

Do you agree that...	Feedback type	*M*(*SD*)	*P*
1. This can help produce an awareness of one’s mental health	General	3.62(0.74)	0.17
	Group	4.08(0.47)	
2. This can help produce a motivation to improve one’s mental health	General	3.31(0.46)	0.02*
	Group	4.15(0.66)	
3. This can help improve one’s interest in mental health	General	3.0(0.68)	< 0.01**
	Group	3.92(0.83)	
4. This can help reduce the reluctance to face one’s mental health	General	2.38(0.62)	< 0.01**
	Group	4.15(0.66)	

1: do not agree, 2: somewhat agree, 3: quite agree, 4: strongly agree, 5: extremely agree

* *p*<.05, ** *p*<.01

##### Improved understanding of one’s mental status

The interviewees expected that students would be able to better understand their conditions. In the case of group-tailored feedback, an integrated explanation was possible by considering the relationship between each MHD. The participants judged that this made it possible for those without a related field of study to more easily understand the information provided (R1,2,6,8,9,10,12,13).

Additionally, it was noted that legibility was improved, and concrete and realistic recommendations were possible by selectively explaining important factors rather than providing personal feedback. These factors ultimately allowed the student to sympathize with the results and to understand them comfortably (R1,4,7,12).

From the survey results, it was evident that group feedback tends be more helpful than individual feedback for individuals to recognize their mental health status (p = 0.17). However, the difference was not statistically significant because it may be individuals who score in the boundary area when the variance is large may have difficulty accepting the feedback (R1,6,8,10,13). Additionally, group feedback is based on the relationship between MHDs. Although it is easy to obtain key points, it could be difficult to grasp the status of a specific MHD (R2,5,8,9,12).

*“In individual feedback, students need to know the features of each mental aspect and synthesize the results themselves. Of course, since you can see each score partially, it has the advantage of knowing your status a little more accurately, but I don’t think they can understand this comprehensively. If it is provided as group feedback, I think it will be helpful to understand because it can explain what kind of connection each measure has.”* (R10)

##### Improved concern about mental health and its management

One factor in which group feedback showed a significant difference from general feedback was increase interest in mental health and motivation to improve mental health (Table 4). According to the Wilcoxon signed-rank test, there was a significant difference in interest in and motivation for improving mental health (*p*<0.05). Comparing each kind of feedback, most of the interviewees often used words such as “interesting”. One of the causes of the result was that concentration and interest increased due to the comprehensive and comfortable understanding of overall mental status (R2,4,6,10).

Another cause was the use of intuitive group names. The interviewees noted that using group names rather than describing the severity of the conditions played an important role in eliciting the interest of the student (R7, 10, 12). In addition, the desire to identify one’s position by categorizing it within one’s social group was seen as another element that increased interest (R2,5,10).

*“Looking at each score alone, it doesn’t draw a picture of what kind of level I am overall, so I think group feedback is much more interesting and easier for general students to take.”* (R6)

This increased interest could lead to motivation to improve mental health. In addition, realistic and tailored recommendations considering overall health status were evaluated to be helpful for motivating self-management of mental health (R2,4,6,7,9).

However, there were opinions that online screening itself could increase one’s interest in mental health (R3,7). It was noted that the motivation for mental health promotion may also vary with mindset rather than the form of feedback (R4,5,6).

*“I am not sure because it seems that the motivation will be determined depending on the current situation of the individual.”* (R7)

##### Reducing reluctance to confront negative result

The item that showed the largest difference in the survey was that feedback could reduce the reluctance to face one’s own mental health (*p*<0.01). Group feedback provides a soft and commentary-like feeling by informing students of their overall situation in an integrated manner (R2,3,6,7,11,12,13). In addition, it also helps individuals feel less problematic because the explanation is given as a general description rather than indicating the severity directly (R2,7,13). The advantage of creating a sense of belonging by categorization into a group within society helped individuals face the results. Social belonging provides a sense of relief even in the face of poor results (R1,2,5,8,10,11).

*“Compared to getting result individually, I would feel less reluctance or sensitivity to accept the results, and I would be able to feel a sense of stability in the presence of similar people as a group, not just me.”* (R8)

## Discussion

The relationship between perfectionism and procrastination was a significant factor in dividing the SDA- with the cluster analysis based on a mild difference in other mental dimensions. The ‘disturbed mind’ group was classified with existing emotional criteria, namely, depression and anxiety, but the distribution of the scores for other MHDs was different from that of the SDA- group. Based on this, the characteristics and suggestions for each group were primarily determined through discussion with experts, and these were evaluated and complemented through interviews with experts.

### Design principles

**Fig. 4:**
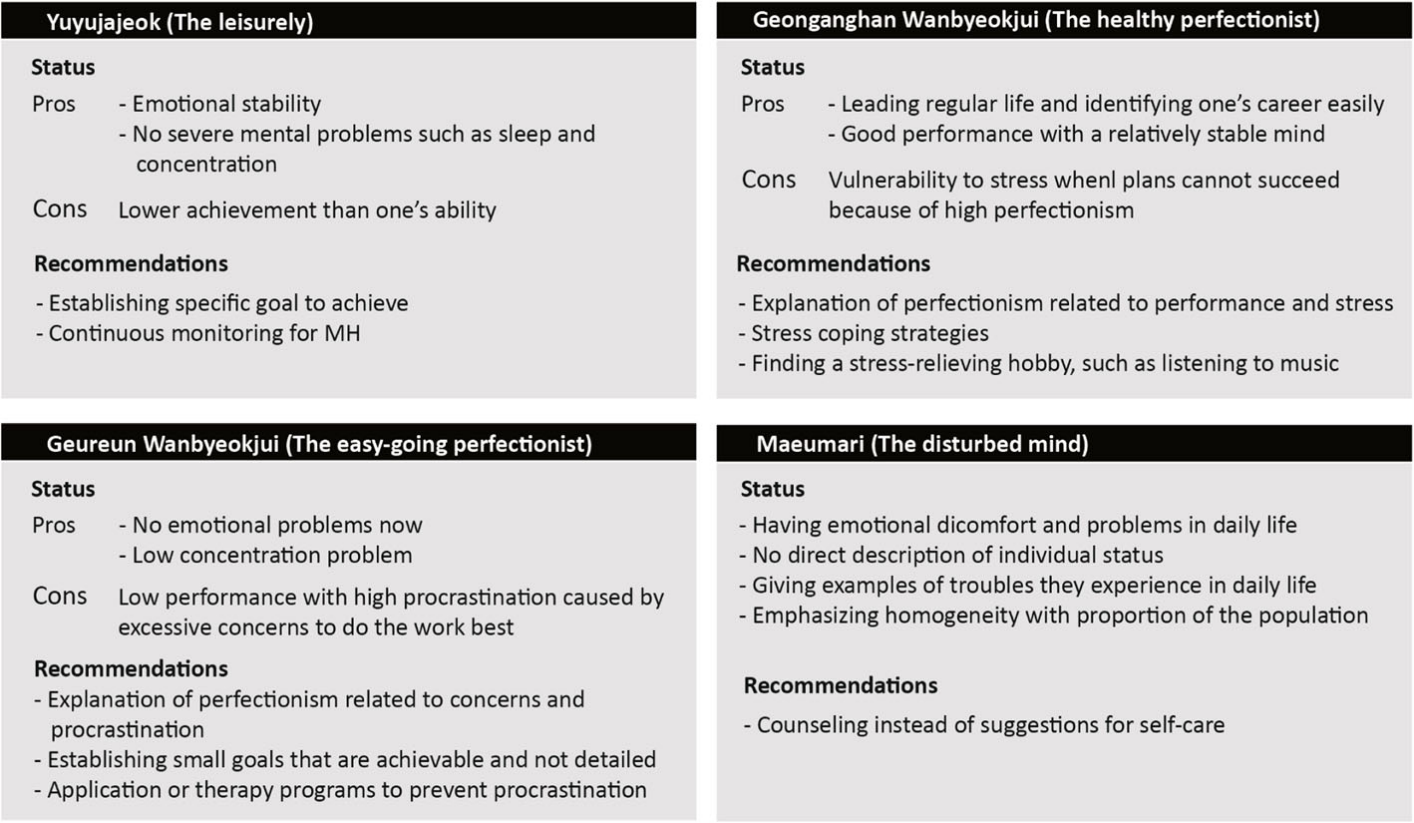
Name and principal elements of the clustered group in group-tailored feedback for university students

The following features and suggestions for each group were summarized (Fig. 4). The names of each group were positively evaluated, but there was a comment that ‘lazy’ can have a negative connotation. Therefore, ‘lazy’ was changed to ‘easy-going’, which shows characteristics of procrastination without a negative expression.

Providing feedback following the mental health screening aimed to increase cognition and motivation to manage one’s own mental health to improve MHL. For this reason, it is essential to increase empathy and the acceptance of the content. Therefore, the following precautions should be taken.

First, it is crucial to highlight the strengths of each groups except ‘the disturbed mind’ group and indicate the weaknesses afterward. By emphasizing strengths, the student can react positively to the following feedback on weaknesses and suggestions. Therefore, the strengths and weaknesses of each group are shown in Fig. 4. The vulnerabilities are organically linked to the recommendations for a healthy mind, so they were selectively presented with concrete examples to induce empathy and motivation to practice interventions rather than presenting various solutions.

Second, the status of each group should be expressed in detail with examples based on the content of questions from questionnaires.

Third, words and expressions should be easy for individuals who do not study in related fields to understand and should be unambiguous. Notably, in the case of ‘the disturbed mind’ group, the misuse of the group name can convey the wrong meaning and produce a negative feeling.

Additionally, definitive expressions should be avoided. In group-tailored feedback, the deviation within a group is not clearly reflected by the group description.

Next, the variability of the features should be mentioned to prevent the student from taking the results as expressing stable characteristics. Mental health can change depending on various contexts in life, so it is recommended to monitor the status steadily with the screening tool.

Finally, it should be stated that users can obtain help from professionals in the university counseling center regardless of the result. It should be implied that obtaining help from professionals can improve one’s mental health because the difficulties one suffers are subjective.

### Implications

The following insights were obtained from the interviews by comparing the group-tailored feedback we developed with existing nonintegrative individual feedback and by supplementing the content. First, group-tailored feedback can help increase the MHL of university students better than general feedback. Group feedback using cluster analysis makes it possible to define mental characteristics by integrating the various MHDs of each group. In this way, students can understand and accept their overall mental features more easily than when interpreting the results based on severity. Unlike the existing method of providing various solutions for each measure separately for mental healthcare, selective recommendations based on the overall condition can be provided, reducing the burden on the individual to think about solutions on their own. In addition, a more extensive consensus is possible based on the group to which the participants belong because the characteristics of this group are communicated rather than classifying individuals according to a constant standard, as done by the general method. The group names collecting the mental features also enabled the results to be intuitive and exciting. As a result, students will be more interested in their mental health status and in managing their mental health themselves with a driving force to practice.

Second, a sense of belonging in one’s society can reduce reluctance to face one’s mental health status. In the case of person-tailored feedback, it is difficult to explain population characteristics by showing results based on the current clinical criteria, and there is a limit to obtaining a sense of belonging because individual scores are objectively evaluated. However, in the case of group-tailored feedback through cluster analysis, it becomes possible to give a sense of belonging because individuals of a similar type are grouped together, and the results are explained within the score distribution of the group. Students may question themselves or feel left out when directly faced with the consequences if they are experiencing depression or anxiety on a clinical level. In that sense, informing people of the features of their group rather than delivering individual scores can help alleviate feelings of isolation. Moreover, it allows negative perceptions of psychiatric examination or treatment to be resolved by reducing the reluctance to face one’s mental health status.

### Strengths and limitations

Our research proposes a new approach for group-tailored feedback based on the clustering of the general mental health profiles of university students. It allows students who do not have severe diseases to also increase their interest in their mental health status and in learning how to care for themselves through help-seeking behavior. Nonetheless,there are several challenges to interpreting the results.

First, the cluster technique is a method of unsupervised learning, so it is difficult to specify the optimal case. We used the k-means clustering method, which showed maximum separation with low within variance (CH score = 91.67), to separate data based on Euclidian distance, so the results were comparatively easy to interpret [47]. However, other solutions can be obtained by adjusting the number of clusters during model training. Therefore, we set the number of clusters using reference metrics such as silhouette score and CH score during modeling and then adjusted the clusters through discussions with experts to draw a meaningful outcome.

In the cluster analysis, the silhouette score (0.32) showed significant variability between the groups, but individuals who score far from the mean within each group may feel heterogeneity. To reduce the within-group deviation, it is necessary to diversify the groups by collecting more samples.

Additionally, the sample size is comparatively smaller (4% of the population) than those of other cross sectional studies using cluster analysis [27, 50–52]. However, our research indicates a methodology for giving group-tailored feedback to a population, so more samples may be necessary to put this research into practice. In this research, professionals expected that the feedback method would be practical, but this expectation was not substantiated empirically by actual users. User studies should be implemented to evaluate the practicality of this method and improve recognition and mental health behavior in future studies.

Last, it cannot monitor symptoms of every mental disorder and specific context of status. This screening tool roughly estimates common mental disorders and other risk factors about mental health that university students can usually have for the sake of usability and accessibility. Our tool does not have diagnostic tools for specific types of disorders and other risk factors in detail. Therefore, our research recommends giving an announcement to contact professionals in university counseling centers for additional explanation or help-seeking.

### Conclusion

This study proposed group-tailored feedback using cluster analysis as a new method to deliver test results following online mental health screening for university students. We grouped students based on mental health score distributions using k-means clustering and determined the mental features and recommendations for mental health. The feedback developed through this process was evaluated based on the multiple dimensions of mental health in an integrated way so that people can easily comprehend the feedback on their current mental status. Furthermore, receiving results based on the group profile to which an individual belongs allows the individual who feel more empathy and a greater sense of social belonging than individuals who receive person-tailored feedback based on the existing cutoff point of each inventory.

University students cannot afford to seek independent mental health, but they have high accessibility to online services. We expect that the developed group-tailored feedback can improve MHL and prevent mental health deterioration when students use online mental health screening services.

## Supplementary Information

**Additional file 1** Normality of collected dataset and distribution of the score of nonclinical group and clinical group.

**Additional file 2** Clustering hyper parameters.

**Additional file 3** Materials of interview, which are the samples of group tailored feedback and general feedback.

## Data Availability

The datasets used and/or analyzed during the current study are available from the corresponding author on reasonable request.

## Abbreviations

MHL: Mental health literacy
MHD: Mental health dimensions
SDA+: with severe depression/anxiety group
SDA-: without severe depression/anxiety group
PHQ-9: Patient health questionnaire-9
GAD-7: Generalized anxiety disorder-7
ASHS: Adolescent sleep hygiene scale
ASRS: Adult ADHD self-report scale
FMPS: Frost multidimensional perfectionism scale
API: Aitken procrastination inventory
Acc: Accuracy
