# Supplementary material for: Group-tailored feedback on online mental health screening for university students: using cluster analysis
Source: BMC Prim Care. 2022 Jan 25;23:19. doi: 10.1186/s12875-021-01622-6 (PMC8790855; doi:10.1186/s12875-021-01622-6)
Supplement: Supplementary file 1 — Additional file 1 Normality of collected dataset and distribution of the score of nonclinical group and clinical group. [file 12875_2021_1622_MOESM1_ESM.pdf]

Additional file 1: Normality of collected dataset and distribution of the score of nonclinical group and clinical group

Table 1 Normality of collected scores of mental health dimensions from participants

	PHQ-9	GAD-7	ASRS	FMPS	API	ASHS
Mental problems	Depression	Anxiety	Attention	Perfectionism	Procrastination	Sleep hygiene
Mean (SD)	7.51 (4.93)	5.25 (4.34)	2.49 (1.56)	109.28 (17.36)	54.28 (12.82)	4.38 (0.5)
Skewness (Z score)	1.13 (5.26)	1.18 (5.43)	-0.05 (-0.27)	0.27 (1.48)	0.17 (0.93)	0.39 (2.1)
Kurtosis (Z score)	1.56 (2.95)	1.27 (2.61)	-0.94 (-4.7)	0.39 (1.19)	-0.57 (-1.95)	0.35 (1.12)

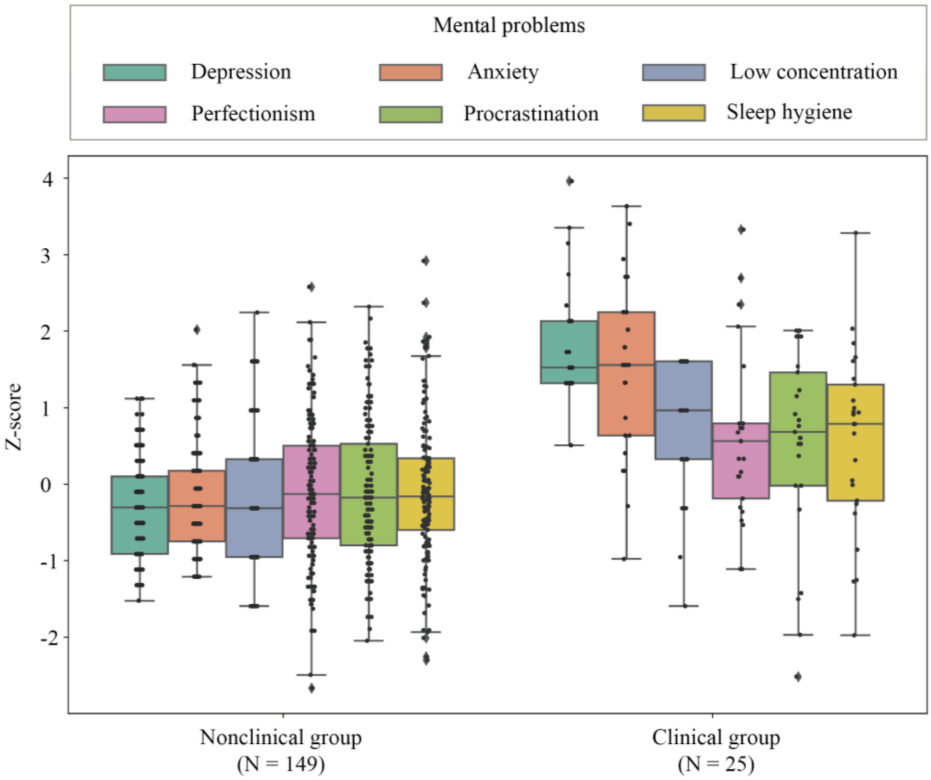

Figure 1 Score distribution of nonclinical group and clinical group
