# Supplementary material for: Group-tailored feedback on online mental health screening for university students: using cluster analysis
Source: BMC Prim Care. 2022 Jan 25;23:19. doi: 10.1186/s12875-021-01622-6 (PMC8790855; doi:10.1186/s12875-021-01622-6)
Supplement: Supplementary file 2 — Additional file 2 Clustering hyper parameters. [file 12875_2021_1622_MOESM2_ESM.pdf]

Additional file 2: Clustering hyper parameters

Table 1 Clustering models for SDA-

Clustering methods	Parameter	Cluster number	Silhouette coefficient	CH score
K-means	-	3	0.32	91.67
Medoid	-	5	0.29	85.75
ANW	-	5	0.3	82.56
ANA	-	5	0.23	32.44
Mean shift	Bandwidth = 19	2	0.35	28.92
DBSCAN	eps = 8	2	0.11	7.4

Table 2 Clustering models for SDA+

Clustering methods	Parameter	Cluster number	Silhouette coefficient	CH score
K-means	-	2	0.44	14.99
Medoid	-	2	0.38	13.98
ANW	-	2	0.50	11.3
ANA	-	2	0.52	7.61
Mean shift	Bandwidth = 27	2	0.50	11.3
DBSCAN	eps = 19	4	0.35	7.21
