# Supplementary material for: Group-tailored feedback on online mental health screening for university students: using cluster analysis
Source: BMC Prim Care. 2022 Jan 25;23:19. doi: 10.1186/s12875-021-01622-6 (PMC8790855; doi:10.1186/s12875-021-01622-6)
Supplement: Supplementary file 3 — Additional file 3 Materials of interview, which are the samples of group tailored feedback and general feedback. [file 12875_2021_1622_MOESM3_ESM.pdf]

Group tailored feedback sample 1. Yuyujajeok (The leisurely)

내담자 정신건강 자가진단 결과

영역 별 역할

그룹 profile

당신은
유유자적 그룹
이시군요!

그룹 상태설명

유유자적 그룹 성향의 사람은 여섯가지 척도에서 모두 안정적인 모습을 보입니다.

제언

결과에서 안정적인 모습이 보인다 하더라도 개인적인 고민이 있을 수 있죠!
정신적으로 문제가 있지 않다 하더라도, 가벼운 문제로도 충분히 상담을 요청 할 수 있습니다.
"사귀는 사람이랑 문제가 있는데.. 어디에 털어놓을 곳도 없고…"
"랩 사람들 때문에 요새 좀 스트레스 받는 것 같아 휴…"
등의 말하기 어려운 문제들도 충분히 상담을 통해 풀어갈 수 있습니다.

유니스트 헬스케어 센터는 언제나 열려있으니 편하게 신청해보세요!

상담 신청 바로가기

다른 그룹들은?

테스트 그룹별 특징

유유자적 그룹 **건강한 완벽주의 그룹** **게으른 완벽주의 그룹** **마음앓이 그룹**

Group tailored feedback sample 2. Geonganghan Wanbyeokjui (The healthy perfectionist)

내담자 정신건강 자가진단 결과

영역 별 역할

그룹 profile

당신은

건강한 완벽주의 그룹

이시군요!

그룹 상태설명

제언

건강한 완벽주의 그룹은 비교적 규칙적이고 자신의 할 일을 잘 찾아가는 유형입니다.
때로는 자신이 만든 다양한 기준에 인해 쉽게 스트레스를 받을 수 있겠군요!

스트레스를 자주 경험하는 상황에서 나만의 스트레스 관리법 하나 정도는 필요합니다.
스트레스 면역이라는 말을 아시나요? 어떠한 스트레스 상황에 직면했을 때,
스트레스에 잘 대처하는 힘을 말합니다.
조그마한 상황에서도 쉽게 스트레스를 받는다면 스트레스 면역력을 끌어올릴 수 있는
'스트레스 면역 훈련'을 시작 해 보세요!
https://www.youtube.com/watch?v=nqqfVjtimxA

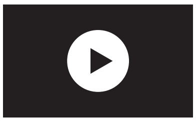

쉽고 짧은 훈련을 통해 나의 스트레스 대처 능력이 키워지는 것을 느낄 수 있습니다.

스트레스를 풀 수 있는 나만의 취미생활도 도움이 됩니다.
바쁜 일상이지만 가벼운 산책이나, 운동 혹은 자기전, 편안한 노래듣기 같은 취미생활을 가꾸어 보아요!
밥먹으면서 보는 예능과 같이 부담스럽지 않은 취미생활도 괜찮아요~

다른 그룹들은?

테스트 그룹별 특징

유유자적 그룹　**건강한 완벽주의 그룹**　**게으른 완벽주의 그룹**　**마음앓이 그룹**

Group tailored feedback sample 3. Geureun Wanbyeokjui (The easy-going perfectionist)

내담자 정신건강 자가진단 결과

영역 별 역할

그룹 profile

당신은

게으른 완벽주의 그룹

이시군요!

그룹 상태설명

제언

게으른 완벽주의 그룹은 완벽주의 성향을 가지고는 있지만 나의 할 일들을 미루는 습관으로 인해 어려움을 경험할 수 있습니다. 이 그룹은 일상생활에서 주의 집중력의 문제와 약간의 정서적 불편감을 느낄 수 있습니다.

완벽주의란 자신만의 높은 기준을 설정하여 높은 성취감을 얻고자 하는 성향인데요, 이러한 완벽주의는 나를 열심히 생활하게 하여 높은 성과를 낼 수 있게끔 해주는 원동력임과 동시에, 내가 그 기대만큼 미치지 못할까봐 시작도 하지 못하게 하는 방해꾼이기도 합니다. 내가 설정한 기준 혹은 다른 사람들의 기대에 의한 기준이 혹시 나에게 불필요한 걱정과 스트레스를 주고 있진 않나요?

게으른 완벽주의 그룹의 스트레스와 과도한 걱정으로 인한 무기력함과 낮은 성취율의 쳇바퀴에서 벗어날 방법으로 규칙적인 생활을 제안합니다.
일단은 현재의 걱정과 미래에 대한 불안을 잠시 접어두고 나만의 규칙을 만들어 봅시다.
예를들어, 아침 8시에 일어나기, 10시에 자기 와 같이 나만의 규칙적인 생활 리듬을 만드는 것입니다.
단순해 보이지만, 이러한 생활습관을 기르는 것만으로도 나에게 성취감을 줄 수 있고, 불필요한 걱정이나 미루는 습관을 개선시킬 수 있으며 불안함과 우울함에도 많은 도움이 될 수 있습니다.

당신의 규칙적 생활습관을 위한, 미루기 습관을 코칭해 줄 어플리케이션을 추천합니다.
생활 리듬의 변화로 건강한 마음을 가꾸어 보세요!

다른 그룹들은?

테스트 그룹별 특징

유유자적 그룹 **건강한 완벽주의 그룹** **게으른 완벽주의 그룹** **마음앓이 그룹**

Group tailored feedback sample 4. Maeumari (The disturbed mind)

내담자 정신건강 자가진단 결과

영역 별 역할

그룹 profile

당신은
마음앓이 그룹
이시군요!

그룹 상태설명

마음앓이 그룹은 최근 다양한 인지나 정서적 불편감을 호소하는 그룹을 말합니다.
모든 척도에서 평균 이상의 점수를 가지는 특성을 가지고 있습니다.

제언

마음 앓이 그룹인 사람들은 정서적 불안감, 수면 등 다양한 경우에서 어려움을 가지고 있을 수 있습니다. 이러한 증상들은 일시적일 수 있습니다. 따라서 주변 사람들에게 마음을 털어놓거나 스스로 치유하면서 해결해 갈 수 있지만, 이러한 증상이 지속되면서 어떻게 해야할 지도 잘 모르겠는 혼란스러운 상황에 직면하기도 합니다.
이러한 어려움이 계속되는 것을 막기 위하여 많은 학생들이 학교 헬스케어 센터를 방문하여 도움을 얻습니다.
혹시 지속적인 힘든 상황에 있었거나, 갑작스런 스트레스 상황에 마음이 많이 어지럽다면, 학교 헬스케어 센터에서 함께 이야기 나누어 보는 것은 어떤가요?

함께 고민을 나누어 보는 시간을 가지기 위한 가벼운 신청서가 아래 링크에 마련되어 있으니, 가볍게 적어주세요.

마음상담 신청하기

혹시 나의 상태가 더 궁금하다면 정서와 관련된 2차 테스트가 준비되어 있으니, 테스트를 해 보셔도 좋습니다:)

정서 설문 하기

다른 그룹들은?

테스트 그룹별 특징

유유자적 그룹　**건강한 완벽주의 그룹**　**게으른 완벽주의 그룹**　**마음앓이 그룹**

General feedback sample 1. General feedback corresponding to 'The easy-going perfectionist' group

내담자 정신건강 자가진단 결과

영역 별 역할

개인 profile

당신은
완벽주의, 지연행동, 불안 에서 경계수준을 나타냅니다.

개인 상태설명
제언

완벽주의가 경계 수준인 경우, 타인에 비해 자신의 기대 수준이 높은 상태를 나타냅니다. 완벽주의는 성과를 높이는 원동력이 되기도 하지만 수준이 높으면 스트레스를 초래하기도 하므로, 평소 산책, 명상과 같은 스트레스 대처법을 익혀 보는 것이 어떨까요?

지연행동이 경계 수준인 경우, 타인에 비해 일을 미루는 경향이 높은 상태를 나타냅니다. 지연행동을 줄이기 위해 일상생활 속에서 작은 생활습관을 만들어 보는 것은 어떨까요? 당신의 규칙적 생활습관을 위한, 미루기 습관을 코칭해 줄 어플리케이션을 추천합니다. 생활 리듬의 변화로 건강한 마음을 가꾸어 보세요!

불안이 경계 수준인 경우, 가벼운 우울증을 나타냅니다.
당장의 전문가와의 상담이 요해지는 것은 아니지만, 힘들면 언제든지 상담을 신청하실 수 있습니다.

General feedback sample 2. General feedback corresponding to 'The disturbed mind' group

내담자 정신건강 자가진단 결과

영역 별 역할

개인 profile	당신은<br>**완벽주의**에서 경계수준을 나타냅니다.<br>**우울, 불안**에서 위험수준을 나타냅니다.
개인 상태설명<br>제언	완벽주의가 경계 수준인 경우, 타인에 비해 자신의 기대 수준이 높은 상태를 나타냅니다.<br>완벽주의는 성과를 높이는 원동력이 되기도 하지만 수준이 높으면 스트레스를 초래하기도 하므로, 평소 산책, 명상과 같은 스트레스 대처법을 익혀 보는 것이 어떨까요?<br><br>우울이 위험 수준인 경우, 우울증의 위험이 높다고 할 수 있습니다.<br>전문가의 상담을 통해 도움을 받아 보는 것은 어떨까요?<br><br>불안이 위험 수준인 경우, 전문가의 상담이 필요한 정도의 불안을 경험하고 있음을 의미합니다.<br>전문가와의 상담을 통해 치료를 받아보는 것이 요해집니다.<br>나의 상태가 더 궁금하다면 정서와 관련된 2차 테스트가 준비되어 있으니, 테스트를 해 보셔도 좋습니다:)<br>정서 설문 하기<br><br>함께 고민을 나누어 보는 시간을 가지기 위한 가벼운 신청서가 아래 링크에 마련되어 있으니, 가볍게 적어주세요.<br>마음상담 신청하기
